# Habitat Radiomics Analysis Based on Non-contrast CT in Differentiation of Parotid Pleomorphic Adenoma and Adenolymphoma

**DOI:** 10.2174/0115734056409272251125042333

**Published:** 2026-01-13

**Authors:** Qifeng Liu, Yaqi Wang, Qi Yao, Bo Duan, Huanyu Chen, Zhimin Ding, Kewu He

**Affiliations:** 1 Department of Imaging Center, Hefei First People’s Hospital, Hefei, Anhui, 230061, China; 2 Department of Radiology, The Yijishan Hospital of Wannan Medical College, Wuhu, Anhui, 241001, China

**Keywords:** Parotid Pleomorphic Adenoma, Adenolymphoma, Habitat, Radiomics, Computed tomograph, Decision curve analysis

## Abstract

**Objective:**

This study aimed to explore the feasibility of habitat radiomics based on Non-Contrast Computed Tomography (NCCT) for differentiating Pleomorphic Adenoma (PA) and Adenolymphoma (AL), and to compare it with both clinical and conventional radiomics models.

**Methods:**

A retrospective collection of clinical and imaging data was conducted on 203 patients who underwent pathology-proven procedures from October 2015 to August 2024 at two hospitals. Tumor Regions of Interest (ROIs) were delineated on NCCT images, and the K-means algorithm was used to jointly cluster the training and validation sets. Radiomics features were extracted, followed by feature selection using the Minimal-Redundancy-Maximal-Relevance (mRMR) and Least Absolute Shrinkage and Selection Operator (LASSO) methods. Univariate and multivariate logistic regression analyses were conducted to identify clinical independent risk factors. The clinical, radiomics, and habitat models were constructed after selection of the clinical and radiomics features. The optimal radiomics model was combined with independent clinical risk factors to develop a nomogram and a combined diagnostic model. The performance of each model was evaluated using the Area Under the Receiver Operating Characteristic (ROC) Curve (AUC), and the DeLong test was used to compare model performance. Calibration curves and Decision Curve Analysis (DCA) were utilized to evaluate model calibration and clinical net benefit, respectively.

**Results:**

Four distinct habitat areas were identified through clustering analysis. The habitat_all model achieved superior predictive performance, with AUCs of 0.903 in the training set and 0.846 in the validation set. This model outperformed the clinical model (training set AUC: 0.837; validation set AUC: 0.823), the conventional intra-tumor radiomics model (training set AUC: 0.845; validation set AUC: 0.840), and each of the four individual habitat models (training set AUCs: Habitat1 = 0.839, Habitat2 = 0.847, Habitat3 = 0.822, Habitat4 = 0.859; validation set AUCs: Habitat1 = 0.823, Habitat2 = 0.840, Habitat3 = 0.827, Habitat4 = 0.842). Furthermore, the nomogram integrating clinical independent risk factors (age and smoking history) with the habitat_all model showed improved predictive performance (AUCs for the training and validation sets were 0.953 and 0.883, respectively) and demonstrated significant clinical net benefit.

**Conclusion:**

Habitat radiomics analysis based on NCCT enables accurate differentiation between PA and AL, providing novel insights for clinical diagnosis and treatment.

## 1 INTRODUCTION

Parotid gland tumors account for 2% to 3% of all head and neck tumors, among which Pleomorphic Adenoma (PA) and Adenolymphoma (AL) are the two common benign types [1, 2]. Although these tumors share similar clinical manifestations, they exhibit distinct biological behaviors, leading to different treatment approaches and prognostic outcomes. PA often progresses to malignancy with non-surgical treatments and has a relatively high postoperative recurrence rate, often necessi-tating superficial or total parotidectomy [3, 4]. Conversely, AL grows slowly and rarely recurs or undergoes malignant transformation after surgery, making clinical follow-up a common recommendation [5, 6]. Therefore, accurate preoperative diagnosis of these two tumor types is crucial for clinical treatment and prognosis.

Imaging examinations, including ultrasonography, CT, and MRI, offer utility for the preoperative differentiation of PA from AL. CT, with its rapid imaging speed, can enhance diagnostic accuracy by demonstrating various enhancement patterns after contrast agent administration, but it carries risks such as adverse reactions to the contrast agent. Ultrasono-graphy has diagnostic advantages for superficial organs but has a relatively limited scanning range, making it challenging to fully capture the overall structure of extensive lesions. MRI provides excellent soft tissue resolution, with different sequences serving diverse functions. Several studies have employed multi-parameter MRI to improve the detection rate of parotid tumors [7-9]. However, these imaging techniques heavily rely on radiologists' subjective experience and judgment. Additionally, as common benign parotid gland tumors, PA and AL exhibit overlapping imaging features, leading to a non-negligible rate of misdiagnosis [10]. Therefore, there is a pressing need to explore methods to improve diagnostic accuracy.

As a non-invasive technique, radiomics enables the extraction of quantitative features from medical images, converting them into interpretable data to facilitate the exploration of tumor tissue composition and biological properties [11, 12]. Currently, CT and MRI-based radiomics methods have demonstrated efficacy in differentiating PA and AL [13, 14]. However, prior studies have focused primarily on analyzing the entire tumor, with limited attention to intratumoral heterogeneity. Recently, habitat analysis has attracted growing interest. Compared to conventional radiomics, habitat analysis subdivides tumors into distinct areas based on internal tissue heterogeneity, enabling character-ization of intratumoral subregions [15]. Several studies have shown that habitat analysis has promising performance in research fields such as lung cancer and brain tumors [16-18]. To date, however, no studies have applied habitat analysis to distinguish between PA and AL. Given the distinct histological characteristics and tissue composition of these two tumor types, habitat analysis may be a valuable tool for improving their differential diagnosis.

This study aims to utilize the habitat analysis technique combined with radiomics to differentiate PA and AL, and to compare with conventional radiomics, focusing on the whole tumor, thereby exploring the feasibility of habitat analysis.

## 2 MATERIALS AND METHODS

### 2.1 Patient Data

We initially collected clinical and imaging data of 337 cases with PA and AL in two hospitals from October 2015 to August 2024. The inclusion criteria were: (1) performance of a Non-Contrast Computed Tomography (NCCT) scan within 14 days prior to surgery; (2) availability of definitive pathological confirmation; (3) no prior radiotherapy or other treatments in the head and neck region. Exclusion criteria were: (1) Incomplete clinical documentation; (2) severe imaging artifacts; (3) tumor maximum diameter less than 5 mm. After applying these criteria, 203 patients were included in the study: 107 with PA (40 males, 67 females) and 96 with AL (86 males, 10 females). Patients were randomly divided into a training set and a validation set at a 7:3 ratio (143 and 60 patients, respectively) (**Supplementary Material**).

### 2.2 CT Examination Methods

Imaging data were acquired using two CT scanners: a Philips Incisive 64-slice CT scanner and a Siemens Somatom Perspective 32-slice CT scanner. The scanning parameters were set as follows: tube current at 140-160mA, tube voltage at 120Kv, pitch at 1.0, slice thickness at 2mm, and scanning range extending from the zygomatic arch to the mandible.

### 2.3 Analysis of Routine Imaging Characteristics

Two radiologists with over 5 years of diagnostic experience, blinded to the results, jointly reviewed the films and reached agreement through discussion. Evaluations included tumour location (left/right), maximum diameter, border (clear/unclear), shape (regular/irregular), involvement of the deep lobe (present/absent, the deep and superficial lobes of the parotid gland are divided by the dorsal-most line of the posterior mandibular vein with the dorsal-most line of the ipsilateral vertebrae [19]), cystic or necrosis areas (present/absent), calcification (present/absent), and ipsilateral enlarged lymph nodes (present/absent, short-axis diameter ≥1cm).

### 2.4 ROI Delineation and Habitat Clustering

NCCT images were exported from the PACS in DICOM format. Resampling the images to 1 mm × 1 mm × 1 mm to eliminate voxel inconsistencies between different scanning machines, and then imported into ITK-SNAP software (version 3.8.0). A radiologist manually delineated the Region of Interest (ROI) slice by slice in a blinded manner. One month later, the prior radiologist and another radiologist randomly selected 40 patients to repeat the delineations. The Intra-class Correlation Coefficient (ICC) was employed to evaluate the inter-observer and intra-observer consistency. ICC values > 0.75 indicated good consistency. Nineteen features extracted from ROI, primarily first-order and textural features such as the Gray-Level Co-occurrence Matrix (GLCM) and the Gray-Level Size Zone Matrix (GLSZM), were used to characterize local features of the whole tumor region for habitat area classification. In previous studies [20-22], it was common to perform cluster analysis on the entire dataset to ensure consistency in habitat definitions, thereby avoiding inconsistencies in habitat labels between the training and test sets due to cluster separation. Therefore, drawing on previous research, the clustering strategy in our study was applied jointly to the training and validation sets. In this study, we used the Davies-Bouldin Index (DBI) to evaluate the effectiveness of the clustering (The calculation formula is 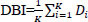). DBI primarily assesses the compactness within clusters and the separation between clusters. A lower DBI indicates better clustering performance. Clustering numbers ranging from 2 to 10 were tested, and the optimal clustering number was determined based on the DBI. ROI delineation and clustering processes are illustrated in Fig. (**1**).

### 2.5 Feature Extraction, Selection, and Diagnostic Model Development

We used the PyRadiomics package in Python [23] to extract radiomics features, including first-order features, shape features, and texture features (texture features include Gray Level Run Length Matrix (GLRLM), Gray Level Dependence Matrix (GLDM), Gray Level Co-occurrence Matrix (GLCM), Gray Level Size Zone Matrix (GLSZM), and Neighborhood Gray Tone Difference Matrix (NGTDM)). Invalid features and those with ICC values < 0.75 were excluded. The remaining raw features were normalized using the Z-score method. Feature dimensionality reduction and selection were conducted using Minimal-Redundancy-Maximal-Relevance (mRMR) and Least Absolute Shrinkage and Selection Operator (LASSO), followed by linear weighting based on feature importance to compute the radiomics score (radscore). Logistic regression classifiers constructed radiomic models for each habitat area, all habitat areas, and the whole tumor region, separately. Clinical models were constructed using logistic regression after identifying clinically independent risk factors via univariate and multivariate analyses. To facilitate clinicians' identification of these two tumour types more intuitively, a nomogram and a combined diagnostic model were developed by integrating independent clinical risk factors with the radscore of the optimal model. The overall experimental workflow was presented in Fig. (**2**).

### 2.6 Statistical Analysis

Statistical analyses and plots were performed using SPSS 26.0 and R software (version 4.2.3). Categorical variables were expressed as the number of cases (n), and differences between groups were compared using the χ^2^ or Fisher's exact test. Continuous variables were expressed as median (quartiles) or mean ± standard deviation. The Kolmogorov-Smirnov test assessed data normality. For normally distributed data, the independent-samples t-test was used; for non-normally distributed data, the Mann-Whitney U test was used. Model performance was evaluated using metrics such as the Receiver Operating Characteristic (ROC) Area Under the Curve (AUC). The DeLong test compared differences between models. Decision Curve Analysis (DCA) assessed clinical net benefit, and calibration curves verified model calibration. *P* < 0.05 was considered statistically significant.

## 3 RESULTS

### 3.1 Clinical and Imaging Data

Table **1** presents the baseline characteristics of all patients and shows no statistically significant differences in clinical and imaging features between the training and validation cohorts (*P* > 0.05). In the training set, univariate logistic regression analysis showed statistically significant differences in age, gender, smoking history, and radscore (*P* < 0.05, Table **2**). Multivariate logistic regression analysis further revealed statistically significant differences in age (*P* = 0.001, OR = 1.073, 95% CI: 1.029-1.120), smoking history (*P* = 0.004, OR = 0.146, 95% CI: 0.039-0.543), and radscore (*P* < 0.001, OR = 2.385, 95% CI: 1.671-3.406) (*P* < 0.05).

### 3.2 Feature Extraction and Selection

**Fig. (3):**
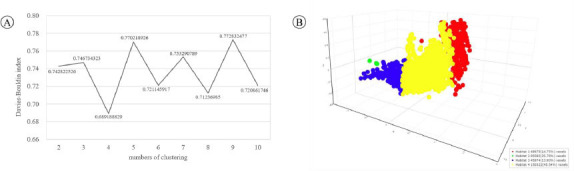
Habitat clustering results. (**A**) The line chart of Davies-Bouldin Index (DBI). The DBI quantified intra-cluster compactness by calculating the average distance between each data point and its respective cluster centroid. A smaller average distance indicated higher intra-cluster density, meaning data points were more tightly grouped around their cluster center. Thus, a lower DBI indicated better clustering performance. The x-axis represented the number of clusters (ranging from two to ten), while the y-axis corresponded to the DBI. The curve in the plot revealed that the DBI fluctuates with varying cluster numbers, reaching its lowest value (0.689188829) at four clusters. This indicated optimal clustering performance at this point, where a favorable balance was achieved between intra-cluster compactness and inter-cluster separation. Based on the DBI, the optimal number of clusters was determined to be four. (**B**) Three-dimensional visualization scatter plot of cluster distribution based on the entire dataset. Distinct colors in the visualization represented the clustering results of different habitats: red corresponded to habitat1, comprising 48,679 voxels (14.75%); green indicated habitat2, with 85,080 voxels (25.78%); blue denoted habitat3, containing 45,974 voxels (13.93%); and yellow represented habitat4, consisting of 150,322 voxels (45.54%). The voxels from each habitat formed well-separated clusters in the three-dimensional space, clearly demonstrating the inherent clustering structure within the 3D feature domain.

**Fig. (4):**
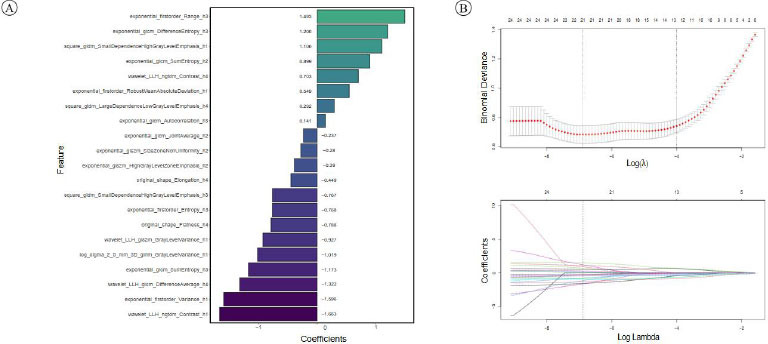
Radiomics feature selection using the Least Absolute Shrinkage and Selection Operator (LASSO) for the habitat_all model and the final feature weights map. (**A**) Distribution of weights for the 21 selected features in the habitat_all model. The x-axis showed the coefficients of selected features. The y-axis showed the 21 features with non-zero coefficients. (**B**) LASSO coefficient profiles showing the feature selection process for the habitat_all model. The regularization path plot shows the selection of the optimal λ (dashed line) via 10-fold cross-validation based on the minimum binomial deviance (a metric quantifying the discrepancy between model-predicted probabilities and observed outcomes). This optimal λ was used to identify the best feature subset comprising 21 features. The coefficient profile plot shows the non-zero LASSO coefficients for 21 features at the optimal λ (dashed line).

Based on the DBI, the optimal number of clusters was determined to be 4 (Fig. **3**). 1834 radiomic features were extracted from the ROI of the whole tumor region. After removing invalid features and retaining those with ICC values > 0.75, 1579 stable radiomics features remained. After feature selection, 9, 9, 9, 10, and 14 radiomics features were obtained in habitat1 to habitat4 and the whole tumor region, respectively. A total of 21 radiomics features were ultimately selected after fusing features across all habitat areas (Fig. **4**). Quantitative comparisons of the 21 radiomics features and visual examples of PA and AL were shown in the supplementary material. Radscore was calculated based on feature weight coefficients (**Supplementary Material**).

### 3.3 Diagnostic Model Development and Performance Assessment

**Fig. (5):**
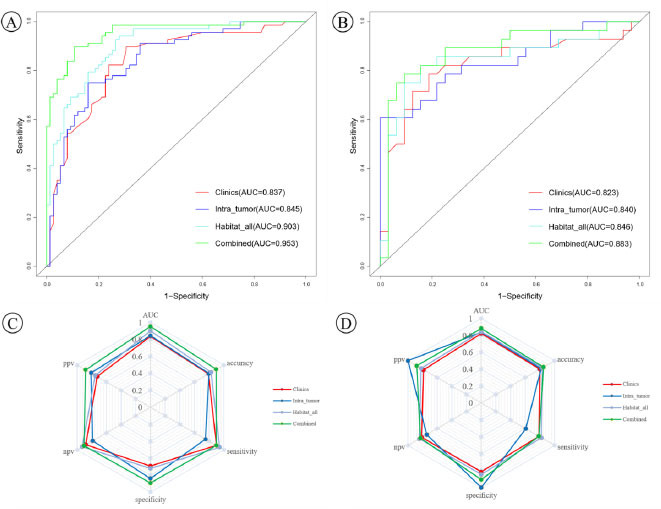
The Receiver Operating Characteristic (ROC) curves and radar charts. ROC curves for the training set (**A**) and validation set (**B**). The red line, blue line, sky blue line, and green line represent the clinical model, intra_tumor, habitat_all, and the combined model, respectively. Area Under the Receiver Operating Characteristic Curve (AUC) for the combined model (AUC for the training set and validation set was 0.953 and 0.883, respectively) was higher than that of the clinics model (AUC for the training set and validation set was 0.837 and 0.823, respectively), intra_tumor model (AUC for the training set and validation set was 0.845 and 0.840, respectively), and habitat_all model (AUC for the training set and validation set was 0.903 and 0.846, respectively). Radar charts for the training set (**C**) and validation set (**D**). The red line, blue line, sky blue line, and green line represent the clinical model, the intra_tumor, the habitat_all, and the combined model, respectively. The evaluation indicators for radar charts include AUC, positive predictive value (ppv), negative predictive value (npv), accuracy, sensitivity, and specificity.

**Fig. (6):**
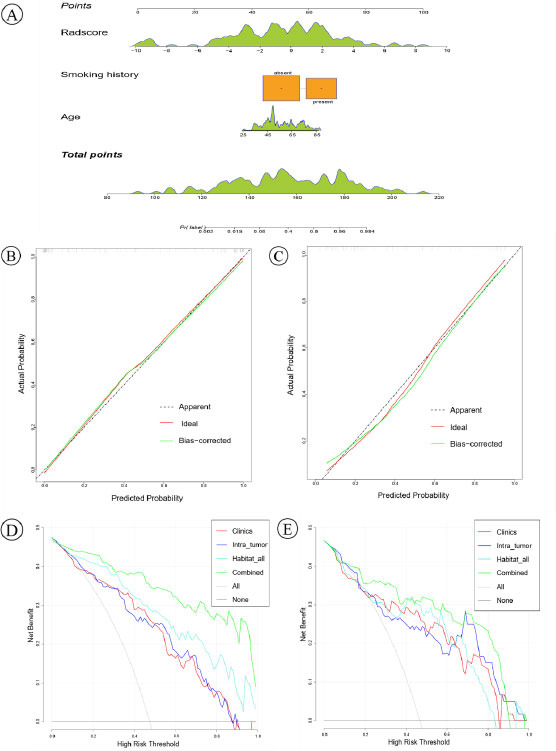
The radiomics nomogram and calibration curves for the combined model, as well as decision curves for the clinical model, intra_tumor model, habitat_all model, and the combined model. The radiomics nomogram, combining Age, Smoking history, and Radiomics score (Radscore), was developed in the training set. The sum of the scores from the corresponding scoring scales for these three factors constitutes the total score. The scale value corresponding to the total score determines the patient's risk probability of AL. Calibration curves of the combined model for the training set (**B**) and validation set (**C**). Calibration curves indicate the goodness of fit of the nomogram. The 45° straight line represents the perfect match between the actual (Y-axis) and nomogram-predicted (X-axis) probabilities. A closer distance between two curves indicates higher accuracy. The combined model fits the ideal curve more closely, indicating it fits well. Decision curves analysis of four models for the training set (**D**) and validation set (**E**). The x-axis indicates threshold probability; the y-axis indicates the net benefit. The red line, blue line, sky blue line, and green line represent the net benefit of the clinical model, intra_tumor, habitat_all, and the combined model, respectively. Across most threshold probabilities, the combined model had a higher overall net benefit in differentiating Pleomorphic Adenoma (PA) from Adenolymphoma (AL).

Based on the above features, habitat1, habitat2, habitat3, habitat4, intra_tumor, and habitat_all models were constructed. The habitat_all model achieved the highest AUC (training set and validation set was 0.903 and 0.846, respectively) (Table **3**, Fig. **5**). The combined model, integrating clinical independent risk factors with the radscore of the habitat_all model, showed enhanced diagnostic performance (training set and validation set was 0.953 and 0.883, respectively) and was significantly better than other models after DeLong test (*p* < 0.05). The radscore of the habitat_all model accounted for a significant proportion in the nomogram (Fig. **6A**). Calibration curves (Fig. **6B**,**C**) and the Hosmer-Lemeshow test indicated that the combined model has a favorable goodness of fit (*P* values of training and validation sets were 0.403 and 0.942, respectively). Decision curve analysis showed the combined model offered higher clinical net benefit (Fig. **6D**,**E**).

## 4 DISCUSSION

Although both PA and AL are benign tumors of the parotid gland, they exhibit distinct biological behaviors, with PA showing a higher propensity for malignant transformation and recurrence compared to AL [3]. Accurate preoperative differentiation of these two tumor types is critical for guiding treatment decisions and optimizing patient outcomes. By segmenting tumors into distinct habitats based on their heterogeneity, we found that the habitat_all model (which integrates features from all subregions) exhibited optimal diagnostic performance. This finding suggests that habitat-based analysis has the potential to differentiate these two parotid gland tumor types.

This study found that the age of patients with AL was generally higher than that of patients with PA. Furthermore, there was a significant gender difference between the two groups. The proportion of female patients in the PA group was 62.6%, whereas in the AL group it was only 10.4%. Additionally, the proportion of patients with a smoking history was higher in the AL group than in the PA group (60.4% *vs*. 21.5%). These findings are consistent with previous studies [13, 14, 24]. We speculated that these differences might be due to the fact that smoking is relatively less common in females, and that irritants in cigarette smoke stimulate metaplasia of parotid glandular and lymphatic tissues, triggering immune responses and delayed-type hypersensitivity reactions, ultimately leading to the development of AL [2, 25]. In terms of imaging manifestations, the proportion of cystic or necrotic areas was slightly higher in PA than in AL (24.3% *vs*. 21.9%), possibly due to fundamental differences in tissue composition: PA is primarily composed of mucus, epithelial cells, and cartilage tissue [26], whereas AL mainly consists of lymphoid mesenchyme and glandular epithelium, with an intercellular matrix rich in lymphocytes [27].

Radiomics is capable of extracting various quantitative features from medical images using high-precision algorithms and techniques [11, 12]. Several studies have demonstrated the excellent performance of radiomics in differentiating PA and AL [13, 14, 28]. Hu *et al.* [13] found that a radiomics model based on T2WI combined with clinical factors could improve the diagnostic efficacy for distinguishing PA from AL (AUCs in the training and validation sets were 0.962 and 0.934, respectively). In another study, Chen *et al.* [28] used enhanced CT radiomics to distinguish three benign tumors of the parotid gland; they found that plain scan, arterial phase, venous phase, and integrated radiomics signature models could effectively differentiate PA from Basal Cell Adenoma and Warthin Tumor. Thus, radiomics holds significant advantages in differentiating PA and AL. The conventional intra-tumor model based on the whole tumor in our study also performed well (AUC was 0.845 and 0.840 in the training and test sets, respectively). However, it should be noted that our study used NCCT exclusively to develop a radiomics model, which has limitations. Nevertheless, NCCT does not require the administration of iodinated contrast agents, thus avoiding the risks associated with them, making its clinical application broader and safer.

Unfortunately, treating the tumor as a whole for radiomics analysis may overlook the impact of intratumoral heterogeneity on results. Recently, habitat analysis techniques have garnered increasing interest. Habitat imaging identifies distinct tumor microenvironments using quantitative imaging techniques, providing insights into tumor phenotype and microenvironment interactions [29]. By dividing the tumor into distinct fragments or subregions based on pixel and feature similarities and differences, habitat analysis is expected to improve the accuracy of study results [15, 16]. Prior investigations have established the clinical utility of habitat radiomics in tumor differential diagnosis, treatment response evaluation, and prognostic assessment [30-32]. Bi *et al.* [33] predicted platinum resistance in high-grade serous ovarian cancer based on a multicenter database, finding the habitat model (AUC = 0.710) outperformed both radiomics model (AUC = 0.640) and deep learning model (AUC = 0.603), with the efficacy of model further improved by combining habitat radiomics features with clinically independent predictors (AUC = 0.721). Our findings align closely with theirs: the habitat_all model outperformed the conventional intra_tumor model, and diagnostic performance was further enhanced by combining it with clinically independent risk factors. The conventional intra-tumor model failed to demonstrate significant diagnostic advantages in our study. We hypothesize that analyzing the tumor as a whole may reduce discriminatory power by averaging out the distinct biological features of different tissue components. In contrast, habitat-based analysis preserves these regional variations, thereby better capturing the tumor's inherent heterogeneity.

In the quantitative analysis of radiomics features of habitat4, the PA showed relatively higher values for Elongation, Contrast, and DifferenceAverage—metrics that quantify tumor shape and the degree of variation in pixel intensity between local regions and their adjacent areas [34, 35]. Specifically, higher Elongation values indicate a more regular tumor shape and sharper edges, while elevated Contrast and DifferenceAverage values further corroborate the clarity of the tumor boundary [34, 35]. These features align well with the pathological manifestations of PA, including its expansile growth pattern, morphological regularity, and fibrous capsule, which radiologically manifest as a well-defined, sharply marginated mass [36]. Conversely, the AL group showed lower values for the aforementioned features, reflecting its pathological traits: lack of a capsule, irregular morphology, multilobulated appearance, and inflammatory infiltration of surrounding tissues [37]. These factors collectively contribute to the blurred boundary between AL and normal tissue [37]. Therefore, we hypothesize that habitat4 may correspond to the interface region between the tumor and normal parotid gland tissue. This finding further suggests that, while both PA and AL are benign tumors, AL exhibits a more pronounced “invasive” tendency in terms of biological behavior compared to PA [5, 6]. In the analysis of habitat1, we found that both PA and AL exhibited low levels of GrayLevelVariance and Variance—metrics that primarily assess the dispersion of pixel intensity values around the mean and the degree of gray-level variation [34, 35]. Lower values indicate greater homogeneity within the region [34, 35]. Given that AL contains cystic components composed of homogeneous fluids and PA possesses uniform mucoid or chondroid matrix components [26-37], we speculate that habitat1 may represent homogeneous cystic regions in PA and loose matrix areas in AL, respectively. Furthermore, higher Range and Entropy values were observed in AL compared to PA within habitat3. These two metrics reflect the difference between the maximum and minimum pixel intensity values in an image and the randomness of image texture, respectively [34, 35]. Higher values indicate the presence of both extremely bright and dark structures within the region, along with a more disordered texture [34, 35]. These quantitative features may be associated with the solid components of AL, namely the complex microenvironment formed by the intermingling and interleaving of epithelial cells and lymphocytes, interspersed with microvessels and microcalcifications [38], which could explain the extreme heterogeneity of its texture. In contrast, PA contains relatively uniform epithelial cells [37], leading us to hypothesize that habitat3 may be closely related to the solid components of the tumor. Finally, in habitat2, we found that the feature values in this region were close to the average in both PA and AL, with minimal differences between the two, suggesting an intermingled interface among different core components in both PA and AL. Therefore, we speculate that habitat 2 may represent the transitional region between cystic, mucoid, or chondroid matrix components and solid tumor components. This quantitative analysis based on habitat radiomics may be helpful in demonstrating the internal heterogeneity of PA and AL.

Our study used the K-means algorithm for clustering analysis, which maintains a relatively stable spatial distribution across different habitat regions, thereby ensuring the accuracy of the clustering results. However, clustering was performed jointly on both the training and validation sets in this study. While this approach ensures all patients share identical cluster centers, it introduces a risk of data leakage. This may lead to overestimated model performance, ultimately compromising the reliable evaluation of the model’s generalizability.

There were some limitations in our study. Firstly, as a retrospective study with a small sample size, it was inevitably subject to selection bias. Multi-centre studies and external validation cohorts are needed in the future to improve the model's universality and stability. Secondly, tumor ROIs were manually segmented using software, which may have introduced inter-observer variability. The adoption of automated segmentation algorithms in future research could enhance the reproducibility and precision of ROI delineation. Finally, our study did not conduct a comparative analysis with pathological sections; the biological significance of each habitat area remains incompletely characterized. Future studies that integrate histopathological correlation are needed to establish definitive associations between habitat-based radiomic features and the underlying tumor biology.

## CONCLUSION

In summary, our study demonstrated that combining habitat analysis with radiomics effectively distinguishes PA from AL. Specifically, the habitat_all model, incorporating features from all tumor subregions, exhibited robust diagnostic performance. Furthermore, the combined diagnostic model, which integrates clinical independent risk factors and habitat-based radiomic features, achieved optimal diagnostic accuracy. Consequently, habitat radiomics based on NCCT holds significant potential as a preoperative, noninvasive tool for distinguishing PA from AL, thereby providing clinically actionable insights for differential diagnosis and treatment planning.

## Figures and Tables

**Fig. (1):**
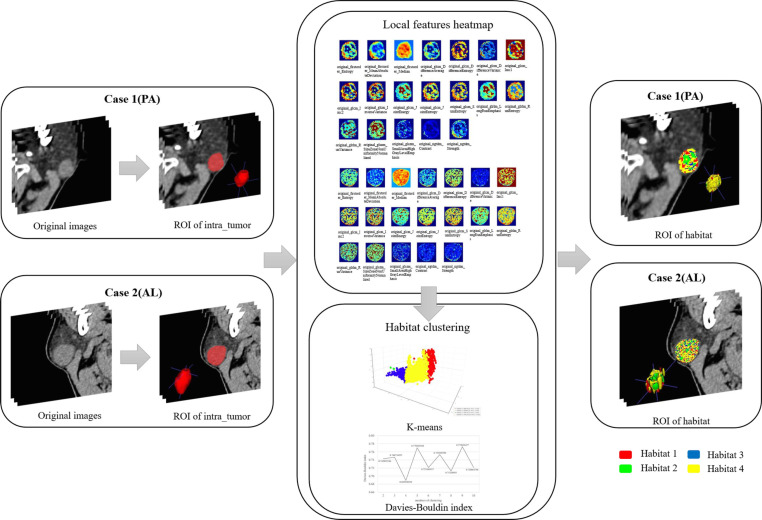
Habitat clustering process. Case 1: a 50-year-old female patient with pleomorphic adenoma (PA); Case 2: a 45-year-old male patient with adenolymphoma (AL). Firstly, the Region of Interest (ROI) was delineated slice by slice, and nineteen features extracted from the ROI were used to characterize local features of the whole tumor region for habitat area classification. The K-means clustering algorithm was applied to all patients, and the Davies-Bouldin Index (DBI) was used to assess clustering performance. Finally, the optimal clustering number was determined based on the DBI.

**Fig. (2):**
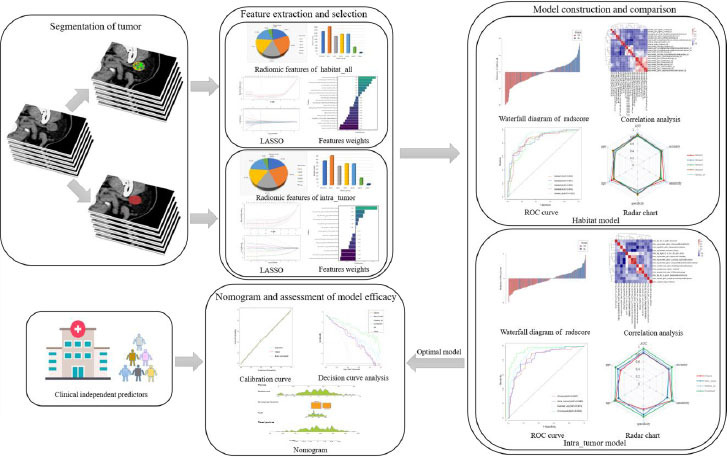
The overall experimental workflow of this study.

**Table 1:** Baseline characteristics of all patients in training and validation sets (*n* = 203).

Clinical and Imaging Characteristics	Training Set (*n*=143)	Validation Set (*n*=60)	*P*
Age ^b^ [*M* (*P*_25_, *P*_75_), year]	53.00(46.00,67.00)	56.00(46.75,68. 50)	0.918
Gender ^a^, *n* (Male / Female)	88/55	38/22	0.810
Smoking history ^a^, *n* (Present/Absent)	58/85	23/37	0.768
Drinking history ^a^, *n* (Present /Absent)	30/113	11/49	0.668
Maximum diameter ^b^ [*M* (*P*_25,_ *P*_75_), mm]	26.00(20.00,35.00)	25.00(18.50,31.50)	0.206
Location ^a^, *n* (Left/Right)	66/77	35/25	0.113
Margin ^a^, *n* (Clear/Unclear)	138/5	54/6	0.062
Shape ^a^, *n* (Regular/Irregular)	103/40	44/16	0.849
Deep lobe involved ^a^, *n* (Present/ Absent)	29/114	15/45	0.456
Cystic or necrotic areas ^a^, *n* (Present/ Absent)	34/109	13/47	0.745
Calcification ^a^, *n* (Present/ Absent)	10/133	1/59	0.180
Enlarged lymph nodes ^a^, *n* (Present/ Absent)	23/120	11/49	0.695

**Note:** ^a^ indicates χ^2^ or Fisher's exact probability method; ^b^ indicates Mann-Whitney U test.

**Table 2:** Comparison of clinical and imaging characteristics of PA and AL group (*n* = 203).

Clinical and Imaging Characteristics	Training Set (*n*=143)			Validation Set (*n*=60)		
	PA(*n*=75)	AL(*n*=68)	*P*	PA(*n*=32)	AL(*n*=28)	*P*
Age ^b^ [*M*(*P*_25_,*P*_75_),year]	49.00 (43.00,57.00)	64.00 (52.25,72.00)	< 0.001	48.50 (42.25,55.50)	66.00 (56.50,70.00)	< 0.001
Gender ^a^ (*n*)			< 0.001			< 0.001
Male	29	59		11	27	
Female	46	9		21	1	
Smoking history ^a^ (*n*)			< 0.001			0.023
Absent	60	25		24	13	
Present	15	43		8	15	
Drinking history ^a^ (*n*)			0.052			0.929
Absent	64	49		26	23	
Present	11	19		6	5	
Maximum diameter ^b^ [*M*(*P*_25_,*P*_75_),mm]	25.00 (18.00,35.00)	28.00 (25.00,34.75)	0.056	23.00 (16.50,30.00)	25.00 (20.00,35.00)	0.151
Location ^a^ (*n*)			0.897			0.726
Left	35	31		18	17	
Right	40	37		14	11	
Margin ^a^ (*n*)			0.669			1.000
Clear	73	65		29	25	
Unclear	2	3		3	3	
Shape ^a^ (*n*)			0.451			0.039
Regular	52	51		27	17	
Irregular	23	17		5	11	
Deep lobe involved ^a^ (*n*)			0.357			0.371
Absent	62	52		22	23	
Present	13	16		10	5	
Cystic or necrotic areas ^a^ (*n*)			0.646			0.967
Absent	56	53		25	22	
Present	19	15		7	6	
Calcification ^a^ (*n*)			0.101			0.467
Absent	67	66		32	27	
Present	8	2		0	1	
Enlarged lymph nodes ^a^ (*n*)			0.347			0.929
Absent	65	55		26	23	
Present	10	13		6	5	
Radscore of habitat_all ^c^ (*x*±*s*)	-2.74±2.90	1.71±2.32	< 0.001	-1.07±2.55	2.12±2.61	< 0.001

**Note:** PA, Pleomorphic Adenoma; AL, Adenolymphoma; ^a^ indicates χ^2^ or Fisher's exact probability method; ^b^ indicates Mann-Whitney U test; ^c^ indicates independent samples t-test.

**Table 3:** Diagnostic efficacy of all models.

Model Name	Group	AUC (95% CI)	Accuracy	Sensitivity	Specificity	Npv	Ppv
Habitat1	Training set	0.839(0.773-0.905)	0.790	0.868	0.720	0.857	0.738
	Validation set	0.823(0.715-0.932)	0.783	0.679	0.875	0.757	0.826
Habitat2	Training set	0.847(0.784-0.909)	0.783	0.750	0.813	0.782	0.785
	Validation set	0.840(0.736-0.945)	0.800	0.679	0.906	0.763	0.864
Habitat3	Training set	0.822(0.752-0.892)	0.762	0.779	0.747	0.789	0.736
	Validation set	0.827(0.722-0.932)	0.750	0.964	0.563	0.947	0.659
Habitat4	Training set	0.859(0.798-0.921)	0.797	0.750	0.840	0.788	0.810
	Validation set	0.842(0.734-0.949)	0.817	0.750	0.875	0.800	0.840
Habitat_all	Training set	0.903(0.854-0.951)	0.825	0.941	0.720	0.931	0.753
	Validation set	0.846(0.737-0.955)	0.833	0.821	0.844	0.844	0.821
Clinics	Training set	0.837(0.770-0.904)	0.790	0.897	0.693	0.881	0.726
	Validation set	0.823(0.707-0.939)	0.800	0.786	0.813	0.813	0.786
Intra_tumor	Training set	0.845(0.781-0.909)	0.797	0.750	0.840	0.788	0.810
	Validation set	0.840(0.737-0.944)	0.817	0.607	1.000	0.744	1.000
Combined	Training set	0.953(0.921-0.985)	0.895	0.897	0.893	0.905	0.884
	Validation set	0.883(0.788-0.977)	0.850	0.786	0.906	0.829	0.880

## Data Availability

All data obtained throughout this study are presented in the manuscript or supporting information. All relevant data are available from the corresponding authors [Z.D] and [K.H] upon reasonable request.

## LIST OF ABBREVIATIONS

PA: Pleomorphic Adenoma
AL: Adenolymphoma
NCCT: Non-contrast Computed Tomography
ROI: Region of Interest
GLCM: Gray-Level Co-occurrence Matrix
GLSZM: Gray-Level Size Zone Matrix
GLRLM: Gray Level Run Length Matrix
GLDM: Gray Level Dependence Matrix,
NGTDM: Neighborhood Gray Tone Difference Matrix
ICC: Intra-class Correlation Coefficient
DBI: Davies-Bouldin Index
mRMR: Minimal-Redundancy-Maximal-Relevance
LASSO: Least Absolute Shrinkage and Selection Operator
Radscore: Radiomics Score
ROC: Receiver Operating Characteristic
AUC: Area Under Curve
DCA: Decision Curve Analysis

## STANDARDS OF REPORTING

STROBE guidelines were followed.
