# Supplementary material for: Habitat Radiomics Analysis Based on Non-contrast CT in Differentiation of Parotid Pleomorphic Adenoma and Adenolymphoma
Source: Curr Med Imaging. 2026 Jan 13;22:e15734056409272. doi: 10.2174/0115734056409272251125042333 (PMC13312404; doi:10.2174/0115734056409272251125042333)
Supplement: Supplementary file 1 [file CMIM-22-E15734056409272_SD1.pdf]

1875-6603/26 Send Orders for Reprints to reprints@benthamscience.net



BENTHAM SCIENCE

CrossMark

# Current Medical Imaging

Content list available at: https://benthamscience.com/journals/cmir

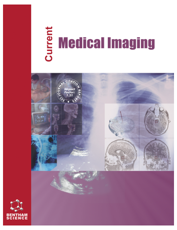


## Supplementary Material

# Habitat Radiomics Analysis Based on Non-Contrast CT in Differentiation of Parotid Pleomorphic Adenoma and Adenolymphoma

Qifeng Liu[1], Yaqi Wang[1], Qi Yao[2], Bo Duan[1], Huanyu Chen[1], Zhimin Ding[2,*] and Kewu He[1,*]

[1]*Department of Imaging Center, Hefei First People's Hospital, Hefei, Anhui, 230061, China*
[2]*Department of Radiology, The Yijishan Hospital of Wannan Medical College, Wuhu, Anhui, 241001, China*

### Radscore of Habitat1

-0.555×exponential_firstorder_Range_h1+0.263×exponential_gldm_LowGrayLevelEmphasis_h1+-1.032×wavelet_LLH_ngtdm_Contrast_h1+-1.596×wavelet_LLH_glszm_GrayLevelVariance_h1+1.461×exponential_firstorder_RobustMeanAbsoluteDeviation_h1+0.714×original_shape_Maximum2DDiameterColumn_h1+0.165×exponential_glcm_Idmn_h1+0.352×square_glszm_LargeAreaLowGrayLevelEmphasis_h1+-0.783×log_sigma_3_0_mm_3D_firstorder_InterquartileRange_h1 + -0.763

### Radscore of Habitat2

-0.787×exponential_glszm_HighGrayLevelZoneEmphasis_h2+-1.748×wavelet_LLH_ngtdm_Strength_h2+0.279×exponential_glcm_Autocorrelation_h2+0.307×exponential_glcm_SumEntropy_h2+1.184×exponential_firstorder_Maximum_h2+0.448×wavelet_LHH_ngtdm_Contrast_h2+-0.533×wavelet_HLH_ngtdm_Strength_h2+-0.523×exponential_firstorder_Entropy_h2+0.855×lbp_3D_m1_firstorder_90Percentile_h2+-0.715

### Radscore of Habitat3

-0.745×exponential_gldm_LowGrayLevelEmphasis_h3+-1.093×wavelet_LLH_glcm_DifferenceAverage_h3+-0.22×exponential_glcm_Idm_h3+1.324×exponential_firstorder_Range_h3+-0.749×exponential_glcm_SumEntropy_h3+1.602×exponential_ngtdm_Coarseness_h3+0.517×log_sigma_3_0_mm_3D_glcm_Id_h3+0.149×wavelet_LLH_ngtdm_Contrast_h3+0.836×exponential_glcm_DifferenceEntropy_h3 + -0.418

### Radscore of Habitat4

0.408×exponential_glcm_MaximumProbability_h4+-0.339×wavelet_HLH_firstorder_90Percentile_h4+-0.449×original_shape_Elongation_h4+0.273×square_glcm_Autocorrelation_h4+0.001×exponential_glszm_LowGrayLevelZoneEmphasis_h4+-0.065×square_gldm_LargeDependenceLowGrayLevelEmphasis_h4+0.491×lbp_3D_k_glszm_SmallAreaLowGrayLevelEmphasis_h4+0.372×wavelet_LLH_glcm_Idm_h4+0.736×exponential_firstorder_InterquartileRange_h4+-0.776×log_sigma_3_0_mm_3D_firstorder_InterquartileRange_h4 + -0.378

### Radscore of Habitat_all

-0.237×exponential_glcm_JointAverage_h2+0.899×exponential_glcm_SumEntropy_h2+1.493×exponential_firstorder_Range_h3+-0.449×original_shape_Elongation_h4+-0.927×wavelet_LLH_glszm_GrayLevelVariance_h1+1.206×exponential_glcm_DifferenceEntropy_h3+-1.596×exponential_firstorder_Variance_h1+-0.39×exponential_glszm_HighGrayLevelZoneEmphasis_h2+0.703×wavelet_LLH_ngtdm_Contrast_h4+0.141×exponential_glcm_Autocorrelation_h3+-1.663×wavelet_LLH_ngtdm_Contrast_h1+0.549×exponential_firstorder_RobustMeanAbsoluteDeviation_h1+-1.322×wavelet_LLH_glcm_DifferenceAverage_h4+-0.768×exponential_firstorder_Entropy_h3+-0.767×square_gldm_SmallDependenceHighGrayLevelEmphasis_h3+-0.28×exponential_glszm_SizeZoneNonUniformity_h2+1.106×square_gldm_SmallDependenceHighGrayLevelEmphasis_h1+-0.788×original_shape_Flatness_h4+-1.173×exponential_glcm_SumEntropy_h3+-1.019×log_sigma_2_0_mm_3D_glrlm_GrayLevelVariance_h1+0.292×square_gldm_LargeDependenceLowGrayLevelEmphasis_h4 + -0.865

### Radscore of Intra_tumor

-0.34×intra_lbp_3D_k_glszm_SizeZoneNonUniformity+-0.181×intra_lbp_3D_k_ngtdm_Busyness+0.503×intra_exponential_glszm_LowGrayLevelZoneEmphasis+0.021×intra_wavelet_LLH_ngtdm_Contrast+-0.017×intra_log_sigma_3_0_mm_3D_glcm_Imc2+0.433×intra_exponential_glcm_MaximumProbability+0.138×intra_logarithm_gldm_DependenceVariance+-0.829×intra_wavelet_LLH_gldm_SmallDependenceEmphasis+1.242×intra_exponential_firstorder_Range+-0.228×intra_exponential_glszm_GrayLevelNonUniformity+0.173×intra_square_glrlm_LongRunLowGrayLevelEmphasis+-0.826×intra_original_shape_Flatness+-0.4×intra_wavelet_LLH_glcm_DifferenceAverage+-0.884×intra_exponential_glszm_GrayLevelNonUniformityNormalized + -0.213

DOI: 10.2174/0115734056409272251125042333, 2026, *22*, e15734056409272

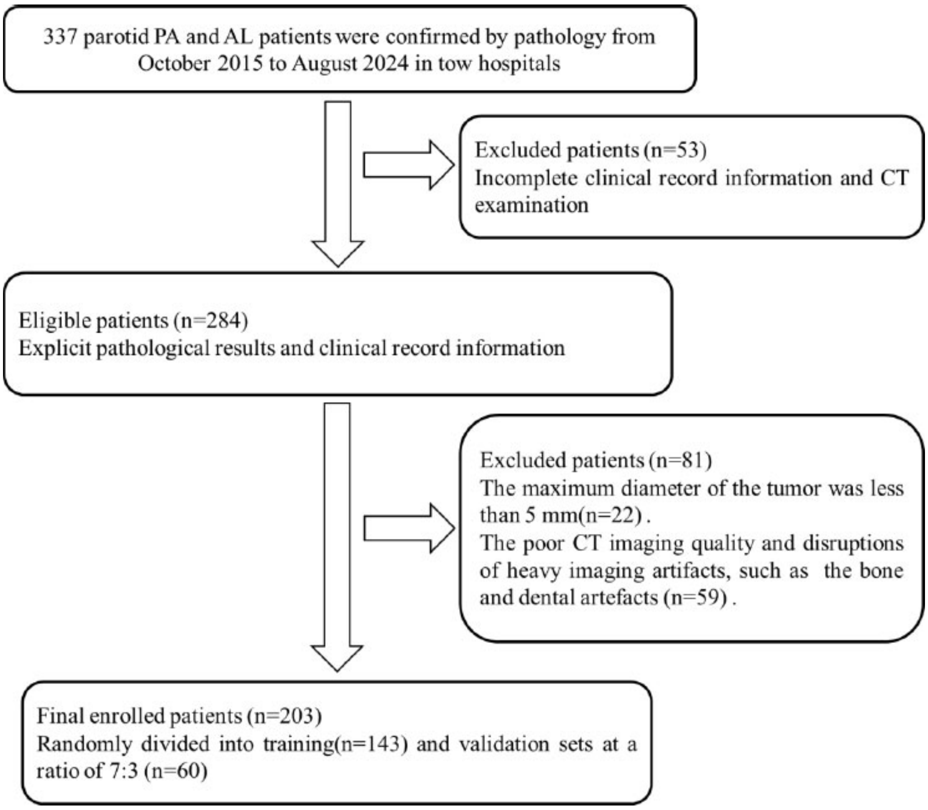


**Fig. (S1).** Flow diagram of the patients inclusion and exclusion process.

**Supplementary Table 1. The quantitative differences comparison of 21 radiomics features between PA and AL.**

| Habitat | Feature Name | PA | AL | *P* value |
|---|---|---|---|---|
| **Habitat1** | wavelet_LLH_glszm_GrayLevelVariance_h1 | 0.145±1.144 | -0.286±0.180 | <0.001 |
| | exponential_firstorder_Variance_h1 | -0.024±1.147 | 0.001±0.230 | <0.001 |
| | wavelet_LLH_ngtdm_Contrast_h1 | 0.104±1.170 | -0.171±0.106 | <0.001 |
| | exponential_firstorder_RobustMeanAbsoluteDeviation_h1 | -0.286±0.809 | 0.427±1.048 | <0.001 |
| | square_gldm_SmallDependenceHighGrayLevelEmphasis_h1 | -0.209±0.923 | 0.161±0.815 | <0.001 |
| | log_sigma_2_0_mm_3D_glrlm_GrayLevelVariance_h1 | 0.130±1.132 | -0.314±0.451 | <0.001 |
| **Habitat2** | exponential_glcm_JointAverage_h2 | 0.003±0.940 | -0.105±0.898 | 0.046 |
| | exponential_glcm_SumEntropy_h2 | -0.004±0.918 | 0.010±1.183 | 0.022 |
| | exponential_glszm_HighGrayLevelZoneEmphasis_h2 | 0.028±0.992 | -0.084±0.973 | 0.006 |
| | exponential_glszm_SizeZoneNonUniformity_h2 | -0.078±0.862 | -0.002±1.106 | 0.043 |
| **Habitat3** | exponential_firstorder_Range_h3 | -0.349±0.824 | 0.409±0.974 | <0.001 |
| | exponential_glcm_DifferenceEntropy_h3 | -0.022±1.028 | 0.060±1.321 | 0.009 |
| | exponential_glcm_Autocorrelation_h3 | -0.119±0785 | -0.044±0.959 | 0.009 |
| | exponential_firstorder_Entropy_h3 | 0.010±1.061 | -0.069±0.865 | 0.008 |
| | square_gldm_SmallDependenceHighGrayLevelEmphasis_h3 | -0.190±0.922 | 0.156±0.973 | <0.001 |
| | exponential_glcm_SumEntropy_h3 | -0.011±1.050 | 0.059±1.190 | 0.009 |
| **Habitat4** | original_shape_Elongation_h4 | 0.194±0.872 | -0.379±1.046 | <0.001 |
| | wavelet_LLH_ngtdm_Contrast_h4 | 0.088±1.195 | -0.136±0.106 | <0.001 |
| | wavelet_LLH_glcm_DifferenceAverage_h4 | 0.244±1.128 | -0.392±0.378 | <0.001 |
| | original_shape_Flatness_h4 | 0.216±0.863 | -0.331±1.049 | <0.001 |
| | square_gldm_LargeDependenceLowGrayLevelEmphasis_h4 | 0.051±0.989 | -0.162±0.653 | <0.001 |

**Note:** PA, pleomorphic adenoma; AL, adenolymphoma; h1, habitat1; h2, habitat2; h3, habitat3; h4, habitat4.

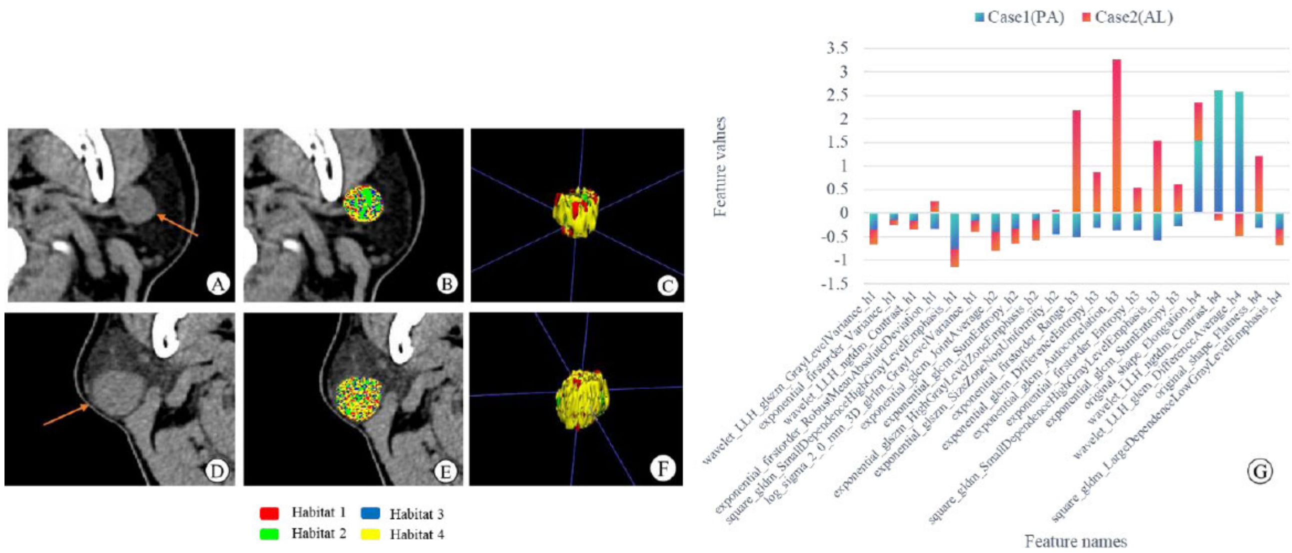


**Fig. (S2).** Visual examples diagram of habitat results and quantitative analysis of radiomics features. Case 1: a 41-year-old female patient with pleomorphic adenoma (PA), the tumor is located in the left parotid gland, with well-defined borders, a round shape, and slightly heterogeneous internal density; case 2: a 45-year-old male patient with adenolymphoma (AL), the tumor is located in the right parotid gland, presenting with poorly defined borders, a slightly flattened shape, and slightly heterogeneous internal density. Figures A-C showed the original image of PA, the ROIs of the four habitats, and the three-dimensional ROI formed by fusion, respectively; Figures D-F show the original image of AL, the ROIs of the four sub-areas, and the three-dimensional ROI formed by fusion, respectively. Among the four divided habitats, red represents habitat1 (h1), green represents habitat2 (h2), blue represents habitat3 (h3), and yellow represents habitat4 (h4). The results of quantitative feature analysis showed that in habitat4, the PA was found to exhibit relatively higher values of Elongation, Contrast, and DifferenceAverage—metrics that quantify tumor shape and the degree of variation in pixel intensity between local regions and their adjacent areas. Higher Elongation values indicate a more regular tumor shape and sharper edges, while elevated Contrast and DifferenceAverage values further corroborate the clarity of the tumor boundary. These features align well with the pathological manifestations of PA, including their expansile growth pattern, morphological regularity, and the presence of a fibrous capsule, which typically manifest as a well-defined, sharp-edged mass on CT. In the habitat1, both PA and AL exhibited low levels of GrayLevelVariance and Variance—metrics that primarily assess the dispersion of pixel intensity values around the mean and the degree of gray-level variation. Lower values indicate greater homogeneity within the region. Furthermore, higher Range and Entropy values were observed in AL compared to PA within habitat3. These two metrics reflect the difference between the maximum and minimum pixel intensity values in an image and the randomness of image texture, respectively. Higher values of them indicate the presence of both extremely bright and dark structures within the region, along with more disordered texture. Finally, in habitat2, the feature values in this region were close to the average in both PA and AL groups, with minimal differences between the two, suggesting the presence of an intermingled interface between different core components within both PA and AL.

© 2026 The Author(s). Published by Bentham Science Publisher.

This is an open access article distributed under the terms of the Creative Commons Attribution 4.0 International Public License (CC-BY 4.0), a copy of which is available at: https://creativecommons.org/licenses/by/4.0/legalcode. This license permits unrestricted use, distribution, and reproduction in any medium, provided the original author and source are credited.

**HOW TO CITE:**
Liu Q, Wang Y, Yao Q, Duan B, Chen H, Ding Z, He K. Habitat Radiomics Analysis Based on Non-Contrast CT in Differentiation of Parotid Pleomorphic Adenoma and Adenolymphoma. Curr Med Imaging, 2026; 22: e15734056409272.
http://dx.doi.org/10.2174/0115734056409272251125042333
